# A Chemical Counterpunch: Chromobacterium violaceum ATCC 31532 Produces Violacein in Response to Translation-Inhibiting Antibiotics

**DOI:** 10.1128/mBio.00948-20

**Published:** 2020-05-19

**Authors:** Gabriel L. Lozano, Changhui Guan, Yanzhuan Cao, Bradley R. Borlee, Nichole A. Broderick, Eric V. Stabb, Jo Handelsman

**Affiliations:** a Wisconsin Institute for Discovery and Department of Plant Pathology, University of Wisconsin−Madison, Madison, Wisconsin, USA; b Department of Molecular, Cellular and Developmental Biology, Yale University, New Haven, Connecticut, USA; c Department of Microbiology, Immunology and Pathology, Colorado State University, Fort Collins, Colorado, USA; d Department of Molecular and Cell Biology, University of Connecticut, Storrs, Connecticut, USA; e Department of Microbiology, University of Georgia, Athens, Georgia, USA; University of Michigan−Ann Arbor

**Keywords:** sublethal concentration antibiotics, two-component regulatory system, *Streptomyces*, microbe-microbe interactions, translation inhibition

## Abstract

Antibiotics produced by bacteria play important roles in microbial interactions and competition Antibiosis can induce resistance mechanisms in target organisms, and at sublethal doses, antibiotics have been shown to globally alter gene expression patterns. Here, we show that hygromycin A from *Streptomyces* sp. strain 2AW. induces Chromobacterium violaceum ATCC 31532 to produce the purple antibiotic violacein. Sublethal doses of other antibiotics that similarly target the polypeptide elongation step of translation likewise induced violacein production, unlike antibiotics with different targets. C. violaceum biofilm formation and virulence against Drosophila melanogaster were also induced by translation-inhibiting antibiotics, and we identified an antibiotic-induced response (*air*) two-component regulatory system that is required for these responses. Genetic analyses indicated a connection between the Air system, quorum-dependent signaling, and the negative regulator VioS, leading us to propose a model for induction of violacein production. This work suggests a novel mechanism of interspecies interaction in which a bacterium produces an antibiotic in response to inhibition by another bacterium and supports the role of antibiotics as signal molecules.

## Abstract (precis)

Secondary metabolites play important roles in microbial communities, but their natural functions are often unknown and may be more complex than appreciated. While compounds with antibiotic activity are often assumed to underlie microbial competition, they may alternatively act as signal molecules. In either scenario, microorganisms might evolve responses to sublethal concentrations of these metabolites, either to protect themselves from inhibition or to change certain behaviors in response to the local abundance of another species. Here, we report that violacein production by C. violaceum ATCC 31532 is induced in response to hygromycin A from *Streptomyces* sp. 2AW, and we show that this response is dependent on inhibition of translational polypeptide elongation and a previously uncharacterized two-component regulatory system. The breadth of the transcriptional response beyond violacein induction suggests a surprisingly complex metabolite-mediated microbe-microbe interaction and supports the hypothesis that antibiotics evolved as signal molecules. These novel insights will inform predictive models of soil community dynamics and the unintended effects of clinical antibiotic administration.

## INTRODUCTION

In many microbial communities, diverse species contribute to complex functions that they cannot perform in isolation (1). Community members also compete with each other (2), in part through production of antibiotics—secondary metabolites that inhibit other community members. The ability for microorganisms to detect and respond to antibiotics is likely to be important for survival and competitiveness in complex communities. The view of antibiotics as tools of both war and peace has evolved over the last 30 years. Although long assumed to be weapons of destruction, it is unlikely that most antibiotics achieve lethal concentrations in the natural world, and thus other roles have been proposed (3–5).

Actinobacteria are prolific producers of secondary metabolites that affect development and secondary metabolism in target bacteria (6, 7). Different classes of secreted secondary metabolites, such as siderophores, biosurfactants, and antibiotics, modulate bacterial interactions. Antibiotics influence intraspecies social behaviors such as biofilm production (8, 9) and contribute to discrimination of kin and non-kin (9). Interspecies interactions mediated by antibiotics include altering virulence (10) and the secreted metabolome (7). In several pathogenic bacteria, sublethal concentrations of antibiotics induce a global transcriptional response, which might be a stress response but might also indicate that antibiotics act as signal molecules (3, 11). A current challenge is to understand how bacteria transduce antibiotic exposure to a targeted transcriptional response. Evidence suggests that antibiotics typically elicit physiological responses through their inhibitory activity rather than by other means such as structural recognition (12, 13). Cellular damage generated by bactericidal antibiotics can induce transcription of stress response genes, but it is less clear how bacteriostatic antibiotics elicit transcriptional changes. The concept of “competition sensing” suggests that some microbes may have evolved the ability to detect a hazard signal by using established stress responses and respond by upregulating production of toxins and antibiotics (14).

*Chromobacterium* species are Gram-negative betaproteobacteria well known for production of violacein, a purple pigment with antimicrobial and antiparasitic activities (15, 16). Violacein has broad antimicrobial activity against diverse Gram-positive bacteria (15), acting by disrupting cell membrane integrity (17). We discovered an interspecies interaction that triggers violacein production in which *Streptomyces* sp. strain 2AW (18) induces the production of violacein in Chromobacterium violaceum ATCC 31532. In the present study, we expand on the understanding of antibiotics as interspecies signals by describing a novel regulatory cascade in C. violaceum ATCC 31532 that enables it to respond to inhibitors of the elongation step of translation by a previously unknown two-component regulatory system.

## RESULTS

### Hygromycin A stimulates production of violacein.

**FIG 1:**
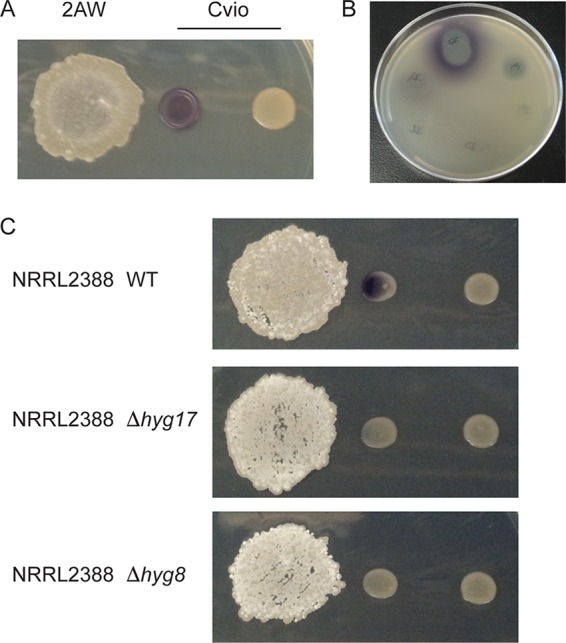
Violacein production by C. violaceum (Cvio) is induced by antibiotics produced by *Streptomyces* spp. (A) C. violaceum growth with *Streptomyces* sp. 2AW (2AW). (B) High-performance liquid chromatography (HPLC) fractions of methanol extract from *Streptomyces* sp. 2AW culture spotted on solid medium spread with C. violaceum. (C) C. violaceum growth with *S. hygroscopicus* NRRL 2388 (NRRL2388) wild type (WT) and two mutants with reduced (Δ*hyg17*) or abolished (Δ*hyg8*) hygromycin A production.

We found that *Streptomyces* sp. 2AW induces the production of violacein by C. violaceum ATCC 31532 (here referred to as C. violaceum) when the bacteria are grown in close proximity (Fig. 1A). Contact is not necessary, suggesting that a diffusible molecule produced by *Streptomyces* sp. 2AW is responsible for triggering the response. Partially purified hygromycin A from *Streptomyces* sp. 2AW at sublethal levels induces violacein production, as does another hygromycin A-producing bacterium, Streptomyces hygroscopicus NRRL 2388 (Fig. 1B and C). Mutations that attenuate or block hygromycin A production (Δ*hyg17* or Δ*hyg8*, respectively [19]) eliminated violacein induction by *S. hygroscopicus* (Fig. 1C). Taken together, these results indicate that hygromycin A is likely responsible for the ability of *Streptomyces* sp. 2AW to induce violacein production in C. violaceum.

### Violacein production is induced by inhibitors of polypeptide elongation.

**FIG 2:**
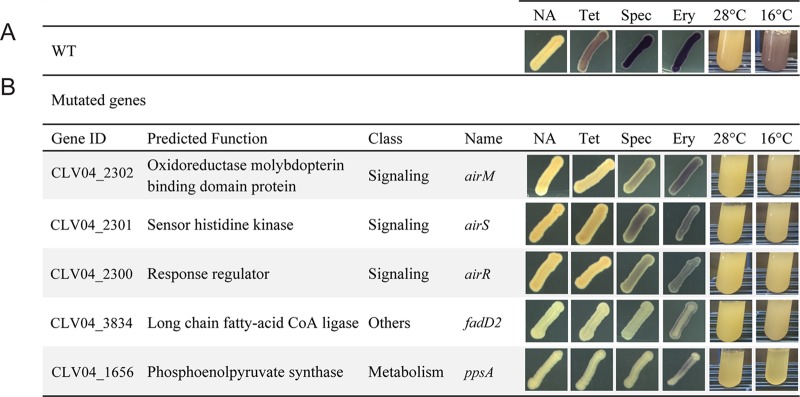
Genes involved in induction of violacein production. (A) Violacein production in response to several inducers in C. violaceum wild type (WT). (B) C. violaceum mutants affected in violacein production in response to all inducers tested. NA, no antibiotic; Tet, tetracycline; Spec, spectinomycin; Ery, erythromycin.

We considered the alternative possibilities that violacein induction could be a response to (i) the hygromycin A molecule specifically, (ii) hygromycin A’s inhibition of translation, or (iii) sublethal antibiosis more generally. To distinguish among these three alternatives, we evaluated diverse classes of antibiotics, including those that block various steps in translation and others that have different cellular targets (see Fig. S1 in the supplemental material). Of the 20 antibiotics tested, 7 induced violacein production in C. violaceum, including blasticidin S, spectinomycin, hygromycin B, apramycin, tetracycline, erythromycin, and chloramphenicol (Fig. 2A; Fig. S1). These antibiotics share two characteristics with hygromycin A, namely, they inhibit growth of C. violaceum, and they block polypeptide elongation during translation, although they belong to different chemical families and inhibit translation by binding to different sites in the ribosomal region responsible for polypeptide elongation. Other antibiotics, including several that block different steps in translation (e.g., kasugamycin, puromycin, and kanamycin), did not induce violacein (Fig. S1).

10.1128/mBio.00948-20.1 FIG S1 Profile of C. violaceum violacein production in response to structurally diverse antibiotics. Antibiotics are classified by cellular target. Download FIG S1, TIF file, 1.6 MB. Copyright © 2020 Lozano et al. 2020 Lozano et al. This content is distributed under the terms of the Creative Commons Attribution 4.0 International license.

To explore whether induction of violacein production is a response to the inhibition of polypeptide elongation, we subjected C. violaceum to cold shock. Sudden decreases in temperature can inhibit polypeptide elongation by generating secondary structures in mRNA (20), and previous studies indicated parallels in responses between translation-inhibiting antibiotics and cold shock (21). We found that rapid transfer of exponential-phase broth cultures from 28°C to 16°C induced violacein production in C. violaceum (Fig. 2A).

### The Air two-component regulatory system is required for the response to translation inhibition.

To identify elements that participate in transducing the stimulus of translation inhibition into the response of induced violacein production, we screened random transposon mutants for loss of this ability. Because hygromycin A is not commercially available, we screened responses to sublethal concentrations of tetracycline, another strong inducer of violacein production (Fig. 2A). Mutants were selected for further characterization if the screen revealed at least two independent mutants with transposon insertions in the same gene, and these genes were different from the quorum-sensing *cviI/cviR* system. To test the role of these genes in the regulatory response to disruption of polypeptide elongation, we further evaluated each mutant’s violacein production when treated with spectinomycin, erythromycin, or cold shock induction at 16°C. We identified five genes that, when disrupted, decrease violacein production in response to each of these stimuli (Fig. 2B). We also identified mutants with altered responses to a subset of treatments (Fig. S2).

10.1128/mBio.00948-20.2 FIG S2 Additional genes involved in induction of violacein production by several inducers. (A) C. violaceum mutants with disrupted violacein production in response to some of the inducers tested. (B) C. violaceum mutants with increased violacein production in response to some of the inducers tested. NA, no antibiotic; Tet, tetracycline; Spec, spectinomycin; Ery, erythromycin; *, transposons in promoter. Download FIG S2, TIF file, 1.4 MB. Copyright © 2020 Lozano et al. 2020 Lozano et al. This content is distributed under the terms of the Creative Commons Attribution 4.0 International license.

### (i) Mutants with similar responses to inhibitors of polypeptide elongation.

**FIG 3:**
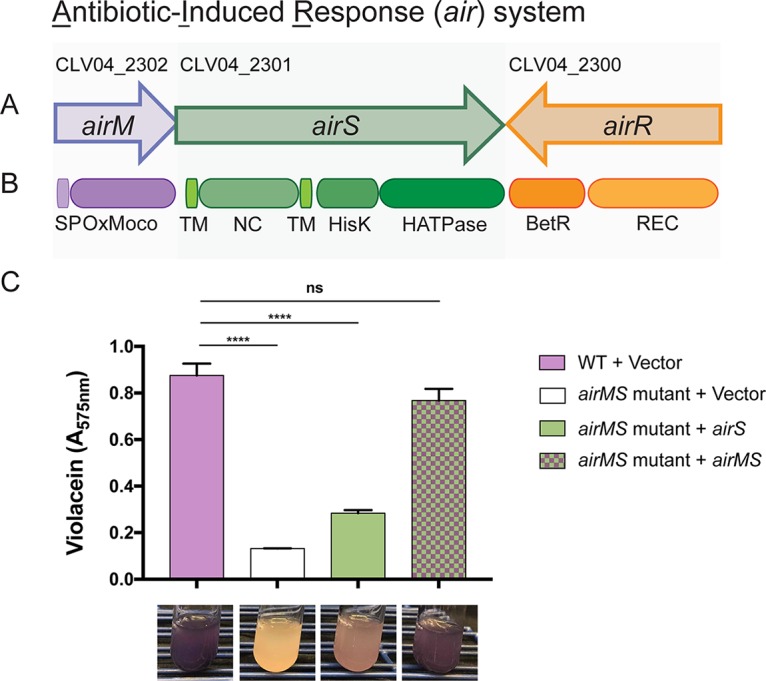
Antibiotic-induced response system. Two-component regulatory system identified by mutant analysis. (A) Gene organization. (B) Functional domains in predicted proteins. SP, signal peptide; OxMoco, oxidoreductase molybdopterin-binding domain superfamily (InterPro accession no. IPR036374); TM, transmembrane domain; NC, noncytoplasmic domain; HisK, signal transduction histidine kinase, dimerization/phosphoacceptor domain superfamily (IPR036097); HATPase, histidine kinase/HSP90-like ATPase superfamily (IPR036890); REC, CheY-like phosphoacceptor receiver domain (IPR001789); BetR, betaproteobacterial transcriptional regulator (IPR013975). (C) Production of violacein in the wild type (WT) carrying an empty vector and in the *airMS* mutant with empty vector or vector carrying *airS* or *airMS*. ****, *P* ≤ 0.0001; ns, not significant (*P* > 0.05).

Strains with mutations in a three-gene cluster encoding a putative two-component regulatory system do not respond to the three antibiotics tested or to cold shock (Fig. 2B). We designated this cluster the antibiotic-induced response (*air*) system, composed of genes that encode proteins predicted to serve as a sensor histidine kinase (AirS), a response regulator (AirR), and an oxidoreductase molybdopterin-binding domain (OxMoco) (InterPro accession no. IPR036374) protein (AirM). The *airS* and *airM* genes appear to be organized in an operon. In many two-component regulatory systems, the sensor and response regulator genes are cotranscribed. However, in this system, *airMS* and *airR* are convergently transcribed (Fig. 3A and B). Notably, three other sensor histidine kinase genes in the genome are similarly arranged near genes encoding an OxMoco domain. Similar systems are also observed in other *Chromobacterium* and *Burkholderia* spp. (Fig. S3). To determine whether *airM* is essential for induction of violacein production, or if the phenotype of the *airM* transposon mutant reflects a polar effect on *airS*, we deleted the *airMS* operon and then complemented the mutant with *airS* or with *airMS*. Complementation with *airMS* restored the response to tetracycline, whereas supplying *airS* alone did not (Fig. 3C), suggesting that *airM* provides an important functional role for the two-component regulatory system.

10.1128/mBio.00948-20.3 FIG S3 Other pairs of genes that encode oxidoreductase molybdopterin-binding domain (OxMoco) (IPR036374) proteins next to a sensor histidine kinase in *Chromobacterium* and *Burkholderia* species genomes. Download FIG S3, TIF file, 0.7 MB. Copyright © 2020 Lozano et al. 2020 Lozano et al. This content is distributed under the terms of the Creative Commons Attribution 4.0 International license.

In addition to the mutants with insertions disrupting the *air* system, we identified strains containing transposon insertions in a phosphoenolpyruvate synthase gene (*ppsA*; CLV04_1656) and a putative long-chain fatty acid coenzyme A (CoA) ligase gene (*fadD2*; CLV04_3834) that likewise display attenuated violacein induction in response to tetracycline or other conditions that inhibit polypeptide elongation (Fig. 2B).

### (ii) Mutants with antibiotic-specific affects.

We also identified several mutants that failed to induce violacein production but only for a specific subset of antibiotics (Fig. S2A). Upon examination, these mutants, including the recently described *cdeR* (CLV04_2412) transcriptional repressor of the *cdeAB*-*oprM* multidrug efflux pump (22), had simply become more resistant to the respective antibiotic, and at higher doses, violacein induction was still evident (Fig. S4).

10.1128/mBio.00948-20.4 FIG S4 Dose-dependent violacein production in two antibiotic-resistant mutants. Survival and violacein production of C. violaceum wild type (WT), C. violaceum with a mutation in a SAM-dependent methyltransferase gene (CLV04_2730), and C. violaceum with a mutation in *cdeR*, a transcriptional repressor of the *cdeAB*-*oprM* multidrug efflux pump system (CLV04_2412) with no antibiotic (A), with erythromycin (μg ml^−1^) (B), with tetracycline (μg ml^−1^) (C), or with spectinomycin (μg ml^−1^) (D). Download FIG S4, TIF file, 2.0 MB. Copyright © 2020 Lozano et al. 2020 Lozano et al. This content is distributed under the terms of the Creative Commons Attribution 4.0 International license.

In addition, strains with mutations in a transcriptional regulator of the GntR family (CLV04_3464) and in an ABC transporter (CLV04_3178) showed a more pronounced induction of violacein in response to the presence of tetracycline, erythromycin, or spectinomycin. Strains with mutations in a putative enoyl-CoA hydratase (*fadB2*; CLV04_1011) likewise showed greater induction of violacein in the presence of the three antibiotics and to the cold shock induction at 16°C, but these mutants also had a higher basal level of violacein production even in the absence of these stimuli (Fig. S2B).

### Additional responses to sublethal concentrations of antibiotics.

**FIG 4:**
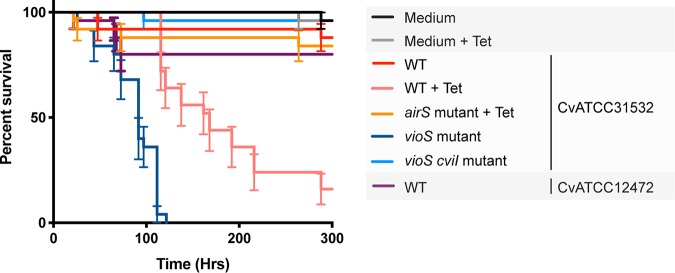
Insecticidal activity of C. violaceum is enhanced by tetracycline. Insecticidal activity of C. violaceum against Drosophila melanogaster with and without a sublethal concentration of tetracycline. C. violaceum (CvATCC31532) wild type (WT), *airS*, *vioS*, and *vioS cviI* mutants, and C. violaceum ATCC 12472 (CvATCC12472) wild type (WT) were evaluated. Tet, tetracycline.

Sublethal concentrations of tetracycline induced phenotypes other than violacein production in C. violaceum. For example, C. violaceum produced biofilms on glass in response to sublethal tetracycline concentrations in an *air*-dependent manner (Fig. S5). We also tested C. violaceum in an oral infection assay with Drosophila melanogaster, which was of interest because of the strain’s close phylogenetic association with Chromobacterium subtsugae, an insect pathogen (Fig. S6). C. violaceum killed D. melanogaster in the presence of tetracycline, but not in its absence, and this tetracycline-induced virulence required the *air* system (Fig. 4). C. violaceum ATCC 12472, a human pathogen that produces violacein, does not show strong insecticidal activity in comparison with C. violaceum ATCC 31532 (Fig. 4). We anticipate that C. violaceum insecticidal activity is independent of violacein, as reported in *C. subtsugae* insecticidal activity against the Colorado potato beetle (23).

10.1128/mBio.00948-20.5 FIG S5 C. violaceum biofilm formation on glass in response to a sublethal concentration of tetracycline. Biofilm formation by C. violaceum wild type (WT) and *vioA*, *airS*, *airR*, *vioS*, and *vioS cviI* mutants without antibiotics or in the presence of tetracycline (0.25 μg ml^−1^). The *vioA* violacein mutant was used as a control for a biofilm visualization without violacein pigmentation. An arrow indicates the location of the biofilm. Download FIG S5, TIF file, 0.8 MB. Copyright © 2020 Lozano et al. 2020 Lozano et al. This content is distributed under the terms of the Creative Commons Attribution 4.0 International license.

10.1128/mBio.00948-20.6 FIG S6 Phylogenetic relatedness of several *Chromobacterium* spp. C. violaceum ATCC 31532 is highlighted in red. Download FIG S6, TIF file, 0.3 MB. Copyright © 2020 Lozano et al. 2020 Lozano et al. This content is distributed under the terms of the Creative Commons Attribution 4.0 International license.

Violacein expression is under the control of the CviI/CviR quorum-sensing system, and it is negatively regulated by *vioS*, an otherwise uncharacterized regulator (24, 25). We tested biofilm formation and insecticidal activity in a *vioS* mutant, which produces violacein constitutively, and in a *vioS cviI* double mutant, which does not produce violacein. Both biofilm production and insecticidal activity are expressed in the *vioS* mutant and not in the *vioS cviI* double mutant, indicating that they are regulated by the CviI/CviR quorum-sensing system and repressed by VioS (Fig. 4; Fig. S5).

### Transcriptional changes in response to sublethal concentrations of antibiotics.

To understand better the physiological response to sublethal concentrations of antibiotics and the role of the *air* system in it, we used global RNA sequencing (RNA-Seq) analysis. C. violaceum wild type (WT) and *airR* mutant were grown both with no antibiotics and challenged separately with tetracycline and spectinomycin, and RNA pools were subjected to RNA sequencing analysis. Each antibiotic induced a distinct but overlapping transcriptional response in the wild type (Fig. S7A). Using Clusters of Orthologous Groups (COG) categories, we analyzed the 640 genes that responded similarly to both antibiotics (Table S1A). Motility genes were enriched among genes that were downregulated in response to tetracycline and spectinomycin (Fig. S7B). Genes involved in translation, ribosomal structure and biogenesis, and secondary metabolite biosynthesis, transport, and catabolism were enriched among those upregulated in response to both antibiotics (Fig. S7B).

10.1128/mBio.00948-20.7 FIG S7 Transcriptome analysis of C. violaceum in the presence and absence of a sublethal concentration of translation inhibitors. The analysis indicates the change in expression from the baseline condition with no antibiotics. (A) Venn diagram of genes differentially expressed by C. violaceum wild type (WT) in streptomycin or tetracycline. (B) COG classification of the differentially expressed genes in streptomycin and tetracycline. Download FIG S7, TIF file, 0.5 MB. Copyright © 2020 Lozano et al. 2020 Lozano et al. This content is distributed under the terms of the Creative Commons Attribution 4.0 International license.

10.1128/mBio.00948-20.8 TABLE S1 (A) Genes expressed differentially in response to streptomycin and tetracycline. (B) Differential gene expression values of the genes identified in the genetic screen. (C) Genes differentially expressed between C. violaceum WT and *airR* mutant in the absence of antibiotics. (D) Genes expressed differentially in response to streptomycin and tetracycline that are modulated by *airR.* Download Table S1, XLSX file, 0.1 MB. Copyright © 2020 Lozano et al. 2020 Lozano et al. This content is distributed under the terms of the Creative Commons Attribution 4.0 International license.

A comparison of the WT transcriptional response with the *airR* mutant response identified 83 genes that were differentially regulated, suggesting that they were directly or indirectly modulated by the *air* system. These transcripts included the violacein gene cluster and two other gene clusters encoding secondary metabolite biosynthetic pathways (Table S1A). Other differentially expressed genes fell in several functional categories with no distinct pattern.

Some genes that were described above as being identified in the transposon mutant screen for altered violacein-induction responses were also found to be regulated in response to tetracycline and spectinomycin. For example, disruption of a gene that encodes a MarR family transcriptional regulator (CLV04_1869) resulted in loss of violacein induction specifically in response to tetracycline (Fig. S2), and this gene was upregulated in response to both spectinomycin and tetracycline (Table S1B). In contrast, disruption of *fadB2*, with a predicted function of an enoyl-CoA hydratase, resulted in a stronger upregulation of violacein production but also higher background expression (Fig. S2), and this gene was downregulated in response to both spectinomycin and tetracycline (Table S1B).

### Mechanisms of violacein induction in response to inhibitors of elongation.

**FIG 5:**
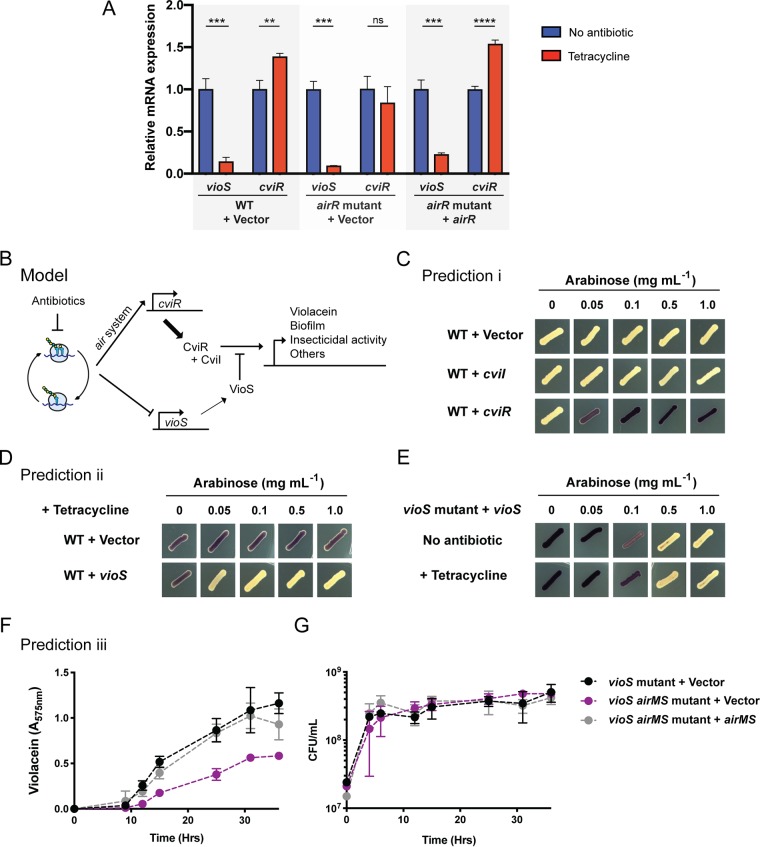
A sublethal concentration of tetracycline bypasses *vioS* repression of violacein production mediated by differential expression of *vioS* and *cviR*. (A) mRNA levels of *vioS* and *cviR* from the C. violaceum wild type (WT) carrying an empty vector, an *airR* mutant carrying an empty vector, and an *airR* mutant carrying a wild-type copy of *airR* in the presence and absence of tetracycline. **, *P* ≤ 0.01; ***, *P* ≤ 0.001; ****, *P* ≤ 0.0001; ns, not significant (*P* > 0.05). (B) Proposed model for violacein induction by translation inhibitors. (C) Overexpression of *cviI* and *cviR* under arabinose regulation. (D) Overexpression of *vioS* under arabinose regulation in the presence of a sublethal concentration of tetracycline. (E) Complementation of *vioS* mutant with *vioS* gene under regulation by arabinose, with and without tetracycline. (F) Violacein production by *vioS* and *vioS airMS* mutant. (G) Growth of *vioS* mutant and *vioS airMS* mutant. Symbol legend applies to panels F and G.

Two known regulators of violacein production, *vioS* and *cviR*, were differentially expressed in the presence of either tetracycline or spectinomycin (Table S1A). VioS is a small protein with no recognizable response domain that represses violacein production (24–26), and CviR is the pheromone-sensing transcriptional activator of a quorum-dependent regulatory system that activates violacein production (25–27). RNA-Seq results for these genes of interest were corroborated and expanded using targeted quantitative reverse transcriptase PCR (qRT-PCR) (Fig. 5A). Transcription of *vioS* is downregulated by sublethal levels of tetracycline in the wild type and in the *airR* mutant, whereas *cviR* is upregulated in the presence of tetracycline but only in the wild type, not in the *airR* mutant (Fig. 5A). The apparent requirement of *airR* for *cviR* induction was confirmed by complementing the *airR* mutant with *airR* in *trans* (Fig. 5A). Thus, *airR* is required for the induction of *cviR* expression in response to tetracycline.

Devescovi et al. recently showed that VioS is sufficient to inhibit expression of the transcriptional promoter upstream of the violacein biosynthetic gene cluster and counteracts activation of this promoter by CviR–*N*-hexanoyl-l-homoserine lactone (C_6_-HSL) in Escherichic coli (24). Although the mechanism of *vioS-*mediated inhibition of the *vioA* promoter is not known, the data suggest that VioS and CviR-AHL compete for the *vioA* promoter or that VioS binds the CviR-AHL complex, thereby blocking its activity. These observations suggest that conditions favoring CviR levels over VioS levels would favor violacein production. We therefore hypothesized that the induction of violacein by sublethal concentration of antibiotics resulted from two independent mechanisms, decreased *vioS* expression and increased *cviR* expression mediated by the *air* system (Fig. 5B).

We drew three predictions from this model (Fig. 5B): (i) increasing the levels of the violacein activator CviR would bypass repression by VioS, (ii) constitutive overexpression of *vioS* would block violacein induction by translation-inhibiting antibiotics, and (iii) the *air* system would mediate the violacein induction response to translation-inhibiting antibiotics even in the absence of *vioS*. We validated predictions i and ii, as follows. Overexpression of *cviR* induces violacein with no antibiotics added, while overexpression of *cviI*, the quorum-sensing autoinducer synthase gene, does not induce violacein production (Fig. 5C). This observation suggests that under these conditions, the quorum-dependent response is limited by CviR levels but not by the autoinducer levels. Also, constitutive overexpression of *vioS* blocks violacein induction in the presence of translation-inhibiting antibiotics (Fig. 5D and E).

To test prediction iii, we first generated the *vioS airMS* double mutant. Unexpectedly, this double mutant produced less violacein than the *vioS* single mutant without tetracycline (Fig. 5F) and without impacting growth fitness (Fig. 5G). The results suggested some activity of the Air system even in the absence of tetracycline. Consistent with this possibility, comparison of the wild-type and *airR* mutant RNA-Seq data in the absence of antibiotics showed that the *air* system affected regulation of at least 15 genes (Table S1C). Thus, the *air* system appears to have some activity even without a translation inhibition signal. Importantly, the data in Fig. 5F show that the *air* system modulates violacein production independently of VioS, as we predicted above.

## DISCUSSION

In this study, we examined an interbacterial interaction mediated by sublethal levels of antibiotics. We found that C. violaceum produces violacein in response to sublethal levels of hygromycin A released from *Streptomyces* sp. 2AW and in response to other structurally diverse bacteriostatic antibiotics that inhibit the elongation step of translation. Genetic analysis in C. violaceum revealed a newly described two-component regulatory complex, the *air* system, that participates in the regulation of violacein production as well as virulence and biofilm production, all of which are regulated by the CviI/CviR quorum-sensing system. Transcriptomic analysis of the wild type and the *airR* mutant showed antibiotic-mediated downregulation of *vioS* and upregulation of *cviR*, revealing a mechanism in which VioS repression of violacein is overcome. Previous work showed that a *vioS* mutant of C. violaceum senses different acyl-homoserine lactones (26), including the one produced by Burkholderia thailandensis (28). Although the interactions with *Streptomyces* and *Burkholderia* both rely on a secreted metabolite regulated by quorum sensing, the mechanisms underpinning their regulation differ. This new interbacterial competition mechanism differs from the previously identified competition strategy of acyl-homoserine lactone-dependent eavesdropping (28) and suggests that C. violaceum can sense and respond to other members of the microbial community in part by using transcriptional regulators that detect inhibitory effects of secondary metabolites produced by their neighbors. Other *Chromobacterium* strains have different regulatory machinery and respond to antibiotics differently from C. violaceum ATCC 31532; therefore, the work reported here pertains only to this strain, and we do not intend to imply that this is a conserved regulatory response among all Chromobacterium violaceum strains.

The idea that antibiotics serve as signals in microbial communities (11) is supported by our findings that sublethal levels of hygromycin A produced by *Streptomyces* sp. 2AW induce violacein production by C. violaceum when the bacteria grow in close proximity. Further experiments are required to determine whether hygromycin A plays a signaling role in natural communities. As observed in human-pathogenic bacteria, sublethal concentrations of antibiotics in C. violaceum also influence social behavior, such as pathogenesis, biofilm formation, quorum sensing, and secondary metabolite production (29). In those systems, it appears that antibiotics function as signals through cellular damage caused by the inducing antibiotic and detected by general stress response networks (14). Among the diverse antibiotics tested, C. violaceum produces violacein only in response to inhibitors of the polypeptide elongation step of translation. Recently, Liu et al. reported a similar phenomenon in which translation inhibitors induce sliding motility in Bacillus subtilis (30). C. violaceum also produces violacein in response to cold shock, providing another example of the long-known parallel between responses to translation-inhibiting antibiotics and cold shock (21). These findings suggest that the activity of the antibiotics, in this case inhibition of the polypeptide elongation step of translation, creates a cellular stress that initiates a signaling cascade.

C. violaceum showed an exquisite response to interference competition mediated by antibiotics that block peptide elongation. Violacein has a broad activity against diverse Gram-positive bacteria (15), and the extremely low solubility of violacein in water enables it to accumulate around the cells. C. violaceum also produces an uncharacterized antibiotic that is active against B. thailandensis and is regulated by quorum sensing (28), illustrating that C. violaceum can mount both broad and specific responses to competitors.

Our work demonstrates a connection between the quorum-sensing regulator VioS, translation perturbation, and a distinctive two-component regulatory system, revealing an unusually refined management of the quorum-sensing system. It makes biological sense that the response to interference competition is regulated by quorum sensing, because antibiotic production at low cell density is unlikely to generate a high enough concentration of antibiotic to inhibit other organisms. The presence of dedicated and specific genetic elements such as *vioS*-*airMSR* differentiates this regulation from the canonical stress responses identified in other competition systems and shows a genetic specialization for detecting and responding to cues from potential competitors.

The *air* system consists of a two-component regulatory system, a sensor (AirS) and a response regulator (AirR), and an oxidoreductase molybdopterin-binding protein (AirM), an unexpected element based on prototypical two-component regulators. In a broad database analysis, we found *airM*-like genes associated with two-component regulatory systems mainly in betaproteobacteria, but the *air* system is the first identified with an associated function. The *air* system is puzzling, because a predicted membrane sensor, AirS, detects a cytoplasmic perturbation of ribosome activity. We hypothesize that the perturbation of actively translating ribosomes would create several cellular changes detected by the *air* system. We cannot infer the nature of the signal detected by AirS, since there is no annotation of known sensor domains in the AirS sequence. The predicted function of AirM, an oxidoreductase protein, suggests that the signal might involve oxidative change. This is compatible with the observed upregulation of the NADH ubiquinone oxidoreductase complex, although expression of oxidative stress pathways did not appear to change (data not shown).

Another potential signal for the *air* system might be alteration in the lipid composition of the membrane. Our genetic screen identified two genes, a long-chain fatty acid CoA ligase gene (*fadB2*) and an enoyl-CoA hydratase gene (*fadD2*), that are homologs of genes that participate in fatty acid catabolism (31). Enoyl-CoA hydratase is downregulated by sublethal concentrations of tetracycline and spectinomycin, and the loss-of-function mutant produces violacein without exposure to antibiotics. The long-chain fatty acid CoA might scavenge phospholipids associated with the membrane, and the downregulation of the enoyl-CoA hydratase mediated by antibiotics could change the pool of saturated and unsaturated acyls-CoA, thereby altering the composition of the new phospholipids added to the membrane. Notably, mutants in phosphoenolpyruvate synthase, a key enzyme in gluconeogenesis, recapitulate the response of the *air* system. Disruption of glucogenesis might impair the production of glycerol 3-phosphate, a key substrate for the synthesis of new triacylglycerols for manufacturing membrane, when the glucose concentration drops, such as in stationary phase. However, we cannot eliminate the possible interaction of other products of gluconeogenesis with the activity of the *air* system.

The *air* system is needed for maximum violacein production without a sublethal concentration of tetracycline in a mutant lacking the negative regulator *vioS*, indicating that the system is active without antibiotic stress and may have a housekeeping function. This is supported by the differential gene expression of several genes mediated by the *air* system without antibiotics. In addition, the presence of a third element in this two-component system may indicate that the *air* system integrates multiple signals of different cellular pathways, as has been shown in other two-component regulatory systems with auxiliary elements (32).

We hypothesized that C. violaceum co-opts a preexisting signaling network to integrate a response generated by the ribosome perturbation. This could expand the model of “competition sensing,” whereby bacteria adapt not only to general stress response networks but also to transcriptional modulators, such as two-component regulatory systems that respond to any physiological response generated by antibiotics. The ribosome is one of the most common targets for antibiotics (33), and being able to sense inhibition of its function rather than detecting each antibiotic independently might enable C. violaceum to have a single response to many competitors rather than separate responses to individual species. This unique response to inhibition of translation could make C. violaceum ATCC 31532 a useful indicator of the mode of action of new antibiotics.

Our discovery of the antibiotic production of C. violaceum in response to antibiosis was facilitated by the fact that this “chemical counterpunch” (i.e., violacein) is purple. With this fortuity in mind, a central question arising from the current study is whether similar phenomena are widespread but less visible. It seems likely that microbial communities possess less obvious but equally important emergent forms of chemical competition. New approaches and technologies (34, 35) have poised the field for a more comprehensive approach to discovering chemically mediated responses that underpin microbial interactions.

## MATERIALS AND METHODS

### Bacterial strains and culture conditions.

*Streptomyces* sp. 2AW (36), Streptomyces hygroscopicus NRRL 2388 (19), Chromobacterium violaceum ATCC 31532 WT, C. violaceum ATCC 31532 *vioS* (Cv017) (26), C. violaceum ATCC 31532 *vioS cviI* (Cv026) (26), and C. violaceum ATCC 12472 were cultured in LB (10 g liter^−1^ tryptone, 5 g liter^−1^ yeast extract, 10 g liter^−1^ NaCl). *S. hygroscopicus* NRRL 2388 and the corresponding mutants were a gift from Kevin Reynolds at Portland State University. Antibiotics were obtained from Sigma (St. Louis, MO, USA) (ceftazidime, chloramphenicol, erythromycin, fusidic acid, hygromycin B, nalidixic acid, paromomycin, piperacillin, polymyxin B, puromycin, tetracycline, trimethoprim, vancomycin), from RPI (Mt. Prospect, IL, USA) (apramycin, blasticidin S, rifampin, spectinomycin), from American Bio (Natick, MA, USA) (kanamycin), from MP Biomedicals (Santa Ana, CA, USA) (streptomycin), and from Enzo Life Sciences (Farmingdale, NY, USA) (kasugamycin).

### Interspecies interaction assay.

*Streptomyces* sp. 2AW and *S. hygroscopicus* NRRL2388 were spotted on LB plates and incubated for 3 to 5 days, when 5 to 10 μl of C. violaceum liquid culture grown for 16 h at 28°C was spotted on two different positions on the plates. The plates were incubated at 28°C until violacein production in C. violaceum was observed.

### Violacein induction assay.

Fractions of a methanol extract from *Streptomyces* sp. 2AW grown on solid medium (18) were directly tested against C. violaceum. Each fraction was spotted on LB plates, and then 100 μl of C. violaceum liquid cultures grown to an optical density at 600 nm (OD_600_) of ∼4.0 at 28°C was spread over the plates. Plates were incubated for 2 days at 28°C. The following antibiotics were evaluated by directly spotting 10 μl of stock solution on LB plates: apramycin (100 μg ml^−1^), blasticidin S (25 μg ml^−1^), ceftazidime (20 μg ml^−1^), chloramphenicol (34 μg ml^−1^), erythromycin (50 μg ml^−1^), fusidic acid (10 μg ml^−1^), hygromycin B (50 μg ml^−1^), kanamycin (50 μg ml^−1^), kasugamycin (10 μg ml^−1^), nalidixic acid (10 μg ml^−1^), paromomycin (10 μg ml^−1^), piperacillin (50 μg ml^−1^), polymyxin B (50 μg ml^−1^), puromycin (25 μg ml^−1^), rifampin (20 μg ml^−1^), spectinomycin (50 μg ml^−1^), streptomycin (100 μg ml^−1^), tetracycline (10 μg ml^−1^), trimethoprim (5 μg ml^−1^), and vancomycin (10 μg ml^−1^).

### C. violaceum transposon mutagenesis and genetic screen for mutants defective in violacein production.

pSAM_BT21 was generated from pSAM_BT20 (37) by exchanging the ampicillin resistance gene for a kanamycin resistance cassette amplified from pENTR/D-TOPO using primers KanTopo_MluIFor/KanTopo_MluIRev (see Table S2 in the supplemental material) and inserted in the MluI site. C. violaceum and E. coli S17-1λpir with pSAM_BT21 with kanamycin (50 μg ml^−1^) were first grown individually for 16 h at 28°C and 37°C, respectively, with agitation. Cells were washed and resuspended in fresh medium to an OD_600_ of 2.0. One volume of E. coli S17-1λpir with pSAM_BT21 was mixed with 2 volumes of C. violaceum. Cells were harvested (6,000 × *g*, 6 min), resuspended in 100 μl of fresh medium, and spotted on LB plates. The conjugation mixture was incubated at 28°C for 6 h and then scraped and resuspended in 2.5 ml of LB. One-hundred-microliter aliquots were plated on LB containing gentamicin (50 μg ml^−1^), ampicillin (200 μg ml^−1^), and tetracycline (0.125 μg ml^−1^) for selection of C. violaceum transconjugants defective in violacein production. Plates were incubated for 2 days at 28°C.

10.1128/mBio.00948-20.9 TABLE S2 Primers used in this study. Download Table S2, PDF file, 0.1 MB. Copyright © 2020 Lozano et al. 2020 Lozano et al. This content is distributed under the terms of the Creative Commons Attribution 4.0 International license.

For each mutant, 1 ml of liquid culture grown for 16 h was harvested (6,000 × *g*, 6 min), and cells were resuspended in 400 μl of TE (10 μM Tris HCl [pH 7.4], 1 μM EDTA [pH 8.0]). Samples were boiled for 6 min and centrifuged (6,000 × *g*, 6 min), and 2 μl of supernatant was used as a template for DNA amplification. Transposon locations were determined by arbitrarily primed PCR (38), which consisted of a nested PCR using first-round primer GenPATseq1 and either AR1A or AR1B and second-round primers GenPATseq2 and AR2 (Table S2). PCR products of the second round were purified by gel extraction (QIAquick gel extraction kit; Qiagen) and then sequenced using primer GenPATseq2. PCR sequencing was performed by the DNA Analysis Facility on Science Hill at Yale University.

### Assay for violacein production in response to antibiotics and cold shock.

Violacein production by mutants identified as defective for violacein induction in response to tetracycline (0.125 μg ml^−1^) was evaluated in the presence of spectinomycin (2 μg ml^−1^), erythromycin (2 μg ml^−1^), and in liquid cultures at 16°C and 28°C. Dose-dependent responses of violacein production to tetracycline (0.25 to 4 μg ml^−1^), spectinomycin (4 to 64 μg ml^−1^), and erythromycin (2 to 16 μg ml^−1^) were evaluated in several mutants on LB plates. The responses to these antibiotics and cold shock were evaluated visually based on the purple color of violacein.

### Chromosomal deletion of the *airMS* operon.

The *airMS* operon was deleted by allelic exchange and replaced with a chloramphenicol resistance cassette. The *airMS* deletion cassette was constructed by a modified version of overlap extension (OE) PCR (39). Fragments 1 kb upstream and 1 kb downstream of the *airMS* operon were amplified using primers MuCv0535/6_Afor and MuCv0535/6_Arev or primers MuCv0535/6_Bfor and MuCv0535/6_Brev, respectively (Table S2). The resulting products had overlapping homology, and further amplification with primers MuCv0535/6_Afor and MuCv0535/6_Brev resulted in a single combined product of approximately 2 kb representing a fusion of the upstream and downstream sequences. This PCR product was cloned into pENTR/D-TOPO, generating pairMS_ENTR. Primers MuCv0535/6_Arev and MuCv0535/6_Bfor were designed to introduce a SphI site in the overlapping region to allow introduction of a selectable resistance gene. A chloramphenicol resistance cassette was amplified from pACYC184 using primers pACYC184Cm_For/pACYC184Cm_Rev, which contain SphI sites in the 5′ region, and cloned into pENTR/D-TOPO, generating pCm_ENTR. The chloramphenicol cassette was recovered from pCm_ENTR using SphI and cloned between the upstream and downstream regions of the *airMS* operon. A *mob* element was recovered from pmob_ENTR (40) using AscI and cloned into an AscI site in the pENTR backbone, generating pairMS_Cm_mob_ENTR. Conjugation mixtures of C. violaceum and E. coli S17-1λpir carrying the pairMS_Cm_mob_ENTR vector were prepared by following the procedure for generating transposon mutants. Double recombinant C. violaceum transconjugants were selected based on their ability to grow on chloramphenicol (34 μg ml^−1^) and screened for the inability to grow on kanamycin (50 μg ml^−1^). The *airMS* deletion mutant was confirmed by PCR using MuCv0535/6_Afor and MuCv0535/6_Brev and by evaluating violacein production in the presence of tetracycline. The same methodology was used to delete *airMS* in the C. violaceum *vioS* mutant.

### Complementation and overexpression assays.

The broad-host-range expression and arabinose-inducible vector pJN105 was modified by introduction of the chloramphenicol resistance cassette recovered from pCm_ENTR into the SphI site, generating pJN105Cm. *airS* was amplified using primers CV0536_For and CV0536_Rev. *airMS* was amplified using primers CV0535/6_For and CV0536_Rev. *airR* was amplified using primers CviR_For and CviR_Rev. *vioS* was amplified using primers CV1055_For and CV1055_Rev. *cviI* was amplified using primers CviI_For and CviI_Rev, and *cviR* was amplified using primers CviR_For and CviR_Rev. For all these genes, an XbaI site in the 5′ region was added to the forward primer, and a SacI site in the 5′ region was added to the reverse primer, for a directional integration of each gene in front of the *araBAD* promoter in pJN105Cm. Plasmids were transferred to the corresponding host using the same conjugation protocol used for generating the transposon mutants. Genes under the control of the *araBAD* promoter were induced with arabinose (0.05 to 1 mg ml^−1^).

### Drosophila melanogaster oral infection assay.

Canton-S (Cs) flies (wolbachia free) were used as standard wild-type lines. *Drosophila* stocks were maintained at 25°C on cornmeal medium (8 g liter^−1^ agar, 80 g liter^−1^ polenta, 40 g liter^−1^ yeast, 40 g liter^−1^ sucrose, 53.6 ml liter^−1^ Moldex). C. violaceum strains were streaked from frozen glycerol stocks onto LB plates and incubated at 28°C overnight. Isolated colonies were then inoculated into LB medium and cultured at 28°C for 20 h. Cultures were centrifuged (4,000 rpm, 20 min, 4°C). The supernatant was decanted, the pellets were resuspended in the remaining liquid, and the concentrations of the cultures were adjusted to an OD_600_ of 200 (approximately 100-fold concentration of the original overnight culture). For antibiotic treatment, tetracycline (2.5 μg ml^−1^) was added to concentrated cultures immediately before the cultures were fed to the flies.

Adult female flies were starved in empty vials for 2 h at 29°C. Paper filters were placed on top of food medium and 150 μl of a 1:1 mixture of the concentrated pellet (OD_600_ = 200) and 2.5% sucrose was added; for the sucrose-negative control, LB was substituted for the bacterial pellet. The starved flies were transferred into the infection vials and kept at 29°C. Survival was assessed at 2 h postinfection to account for any infection-independent mortality. The number of dead flies per vial was recorded twice per day for approximately 5 days after infection. Potential differences in survival between treatments were analyzed for significance with Kaplan Meier survival analysis using GraphPad Prism software.

### Phylogenetic analysis.

*Chromobacterium* species genomes were recovered from the NCBI database, June 2017 (Table S3). Phylogenomic reconstruction was accomplished using the phylogenetic and molecular evolutionary (PhaME) analysis software (41). PhaME identified single-nucleotide polymorphisms (SNPs) from the core genome alignments, and the phylogenetic relationships were inferred by maximum likelihood using FastTree.

10.1128/mBio.00948-20.10 TABLE S3 *Chromobacterium* species genomes used for phylogenetic reconstruction. Download Table S3, PDF file, 0.1 MB. Copyright © 2020 Lozano et al. 2020 Lozano et al. This content is distributed under the terms of the Creative Commons Attribution 4.0 International license.

### RNA-Seq analysis.

C. violaceum WT and *airR* mutant strains were grown without antibiotics, with tetracycline (0.125 μg ml^−1^) or with spectinomycin (2 μg ml^−1^) in 5 ml of LB at 28°C with agitation in duplicate. RNA samples were prepared from 250 μl of cells grown to an OD_600_ of ∼3.2. Cultures were mixed with 750 μl of TRIzol and incubated at 65°C for 10 min. Samples were frozen at –80°C for 10 min and thawed at room temperature for 15 min. Chloroform (200 μl) was added, and the samples were shaken and incubated at room temperature for 3 min and centrifuged (12,000 × *g*, 15 min, 4°C). The aqueous phase was recovered, mixed with 500 μl of isopropanol, incubated at room temperature for 10 min, and centrifuged (12,000 × *g*, 10 min, 4°C). The pellet was washed with 1 ml of 75% ethanol and air-dried for 10 min, resuspended with 50 μl of RNase-free water, and finally incubated at 60°C for 15 min. DNA was removed from 5 μg of total RNA using the TURBO DNase kit (Invitrogen, Carlsbad, CA, USA).

RNA samples were treated with the Ribo-Zero rRNA removal kit (Illumina, San Diego, CA, USA), cDNA libraries were constructed with an average size of 150 to 200 bp, and they were sequenced in an Illumina HiSeq 2500 paired-end 2 × 75 platform. Library preparation and sequencing were performed by the Yale Center for Genome Analysis. Low-quality sequences were trimmed using Trimmomatic (42). Mapped reads and estimated gene expression levels were calculated using RSEM with a transcript list of open reading frames (ORFs) recovered from the C. violaceum genome (GenBank assembly accession no. GCA_002865685.1) (43). Differential expression was assessed using limma (44) using the “voom” function to model the mean variance relationship from read counts converted to log_2_ counts per million (logCPM). Genes that were not appreciably expressed (transcripts per million [TPM] < 5) were discarded, as recommended (44). Genes with an adjusted *P* value of <0.01 were identified as being differentially expressed.

Genes were identified as having a generalized response to inhibition of translational elongation if they were differentially expressed in the WT in the presence of tetracycline and in the presence of spectinomycin, relative to that with no antibiotic (Table S1A). Genes for which the response to translational inhibition is mediated, directly or indirectly, by the *air* system were identified if they were not differentially expressed in response to tetracycline or streptomycin in the *airR* mutant background (but were in WT), or if these genes were differentially expressed when the WT was compared with the *airR* mutant in the presence of both antibiotics (Table S1D).

### qRT-PCR.

Quantitative reverse transcriptase PCR (qRT-PCR) was used to validate the differential gene expression detected for *vioS* and *cviR* in the RNA-Seq analysis. Primers used are listed in Table S2. C. violaceum WT pJN105Cm, *airR* pJN105Cm, and *airR* pJN105Cm_airR were grown with chloramphenicol (34 μg ml^−1^) and with or without tetracycline (0.125 μg ml^−1^) in triplicate as reported above. Total RNA was recovered and DNase treated in the same manner as the RNA recovered for RNA-Seq analysis. Two hundred nanograms of DNase-treated RNA was reverse transcribed into cDNA using the SuperScript III first-strand synthesis system (Invitrogen). Quantitative PCR was carried out in a 10-μl volume using PowerUp SYBR green master mix (Applied biosystems) with 1 μl of cDNA and 200 nM PCR primers. These reactions were performed using the CFX96 real-time system (Bio-Rad) with the following cycling parameters: 50°C for 10 min, 95°C for 5 min, followed by 40 cycles of 95°C for 10 s and 60°C for 30 s. Reverse transcriptase-minus (RT-minus) template PCRs were included as negative controls to confirm the absence of genomic DNA contamination. Tenfold serially diluted DNA standard curves were included on every plate. Melting curve analyses were done to verify the specificity of the PCR products. Expression levels under each condition were normalized to the *dnaG* housekeeping gene, and the Pfaffl method was used to calculate fold change in gene expression (45). Differences between groups were tested for statistical significance (Student's *t* test) using GraphPad Prism 7 software.

### Characterization of C. violaceum *vioS airR*.

C. violaceum *vioS* pJN105Cm, *vioS airMS* pJN105Cm, and *vioS airMS* pJN105Cm_airMS were grown with chloramphenicol (34 μg ml^−1^) and arabinose (0.2 mg ml^−1^) with agitation for 2 days at 28°C. Samples were withdrawn periodically to evaluate bacterial growth by serial dilution and plating in LB and to quantify violacein production. Violacein was quantified by a crude violacein extraction (46). One-milliliter aliquots of cultures of each strain were centrifuged (14,000 × *g*, 20 min), and cells were resuspended in 1 ml of ethanol to dissolve violacein. Supernatants were recovered after centrifugation (12,000 × *g*, 10 min) and transferred to 96-well plates. Violacein concentration was determined spectrophotometrically at 575 nm in a Synergy HT plate reader (BioTek).
