# Supplementary material for: A Chemical Counterpunch: Chromobacterium violaceum ATCC 31532 Produces Violacein in Response to Translation-Inhibiting Antibiotics
Source: mBio. 2020 May 19;11(3):e00948-20. doi: 10.1128/mBio.00948-20 (PMC7240160; doi:10.1128/mBio.00948-20)
Supplement: TABLE S2 [file mBio.00948-20-st002.pdf]

TABLE S2. Primers used in this study.

Name	Sequence
KanTopo_MluIFor	CACCACGCGTACAGCAAGCGAACCGGAATTG
KanTopo_MluIRev	ACACGCGTCTCATGAGCGGATACATATTTGAATG
GenPATseq1	CTTGGATGCCCGAGGCATAG
GenPATseq2	CTGTACAAAAAAACAGTCATAACAAGCCATG
AR1A	GGCCACGCGTCGACTAGTACNNNNNNNNNNGTAAT
AR1B	GGCCACGCGTCGACTAGTACNNNNNNNNNNGATGC
AR2	GGCCACGCGTCGACTAGTAC
MuCv0535/6_Afor	CACCACATGTGCTGCCGCTTTACCGTTGAC
MuCv0535/6_Arev	CGCGAATTGGCTTGAAACCGATCAGCATGCCATTTCGCCTCCCGCCGA
MuCv0535/6_Bfor	TCGGCGGGAGGCGAAATGGCATGCTGATCGGTTTCAAGCCAATTCGCG
MuCv0535/6_Brev	TAACATGTCGGCAACCAAGCCATGTCATG
pACYC184Cm_For	TAGCATGCGTTTTTATCAGGCTCTGGGAGGC
pACYC184Cm_Rev	CACCGCATGCGATAGAAACAGAAGCCACTGGAGC
pJN105Mob_For	TAGGCGCGCCTGTGGTCAAGCTCGTGGGC
pJN105Mob_Rev	CACCGGCGCGCCCAATTCGTTCAAGCCGAGATCGGC
CV1055_For	CACCTCTAGAAGGAGGGTTTTGCCATGCCTTG
CV1055_Rev	CGAGCTCTTAGAGCACGCGGGTAAGCAG
CviI_For	CACCTCTAGAAGGAGGCTTGAGTGAAAAAGTTCTAC
CviI_Rev	CGAGCTCTCAATGCGAATAATCGTACTCACGCC
CviR_For	CACCTCTAGACAAGGAAGACTCGCTCATGGTGATCTC
CviR_Rev	CGAGCTCTCATTCGTTCGCTACGGTCGAGG
CV0536_For	CACCTCTAGACATCGTGGTGCGTTGATGAGAATTCC
CV0536_Rev	CCGAGCTCTCAGACCAGCTCAACCGGC
CV0535/6_For	CACCTCTAGAGAATCGGCGGGAGGCGAAATGA
CviR_For	CACCTCTAGACAAGGAAGACTCGCTCATGGTGATCTC
CviR_Rev	CGAGCTCTCATTCGTTCGCTACGGTCGAGG
RTCvvioSFor	GCCTTGCATCACCCGCAG
RTCvvioSRev	TGATCCTGGCCGGCCAATTG
RTCvcviRFor	CATGGCCGGTCACATCGAGAC
RTCvcviRRev	CAGTCGCTGGGGTAGCTGAC
RTdnaGFor	TCGCTGGAGCAACTGATGCAAATG
RTdnaGRev	TGGTAGTTGGCGGTGATCGC
