# Supplementary material for: A Chemical Counterpunch: Chromobacterium violaceum ATCC 31532 Produces Violacein in Response to Translation-Inhibiting Antibiotics
Source: mBio. 2020 May 19;11(3):e00948-20. doi: 10.1128/mBio.00948-20 (PMC7240160; doi:10.1128/mBio.00948-20)
Supplement: TABLE S3 [file mBio.00948-20-st003.pdf]

TABLE S3. *Chromobacterium* spp. genomes used for phylogenetic reconstruction.

Name	Strain Name	GenBank Assembly accession
Chromobacterium vaccinii 21-1	211	CP017707.1
Chromobacterium violaceum 16-454	16454	MUKS00000000.1
Chromobacterium violaceum 16-419A	16419A	MUKQ00000000.1
Chromobacterium violaceum 16-419B	16419B	MUKR00000000.1
Chromobacterium violaceum ATCC12472	ATCC12472	AE016825.1
Chromobacterium violaceum ATCC31532	ATCC315322	PKBZ00000000.1
Chromobacterium sp. ATCC 53434	ATCC53434	CP025429.1
Chromobacterium sp. C-61	C61	CAEE00000000.1
Chromobacterium aquaticum CC-SEYA-1	CCSEYA1	MQZY00000000.1
Chromobacterium violaceum strain CV1192	CV1192	CP024028.1
Chromobacterium violaceum strain CV1197	CV1197	CP024029.1
Chromobacterium haemolyticum DSM 19808	DSM19808	JONK00000000.1
Chromobacterium amazonense DSM26508	DSM26508	MKCR00000000.1
Chromobacterium subtsugae F49	F49	JWJN00000000.1
Chromobacterium sp. F49	F492	LQNP00000000.1
Chromobacterium violaceum GHPS1	GHPS1	NHOO00000000.1
Chromobacterium violaceum GN5	GN5	JWPW00000000.1
Chromobacterium haemolyticum H3973	H3973	MUKT00000000.1
Chromobacterium haemolyticum H4137	H4137	MUKU00000000.1
Chromobacterium haemolyticum H5244	H5244	MUKV00000000.1
Chromobacterium violaceum H5524	H5524	MUKW00000000.1
Chromobacterium violaceum H5525	H5525	MUKX00000000.1
Chromobacterium sphagni IIBBL 14B-1	IIBBL14B1	MKCT00000000.1
Chromobacterium sphagni IIBBL 37-2	IIBBL372	MKCS00000000.1
Chromobacterium violaceum L_1B5_1	L1B51	JYGI00000000.1
Chromobacterium sp. LK1	LK1	LDUI00000000.1
Chromobacterium sp. LK11	LK11	LDUR00000000.1
Chromobacterium violaceum LK15	LK15	LDUT00000000.1
Chromobacterium violaceum LK17	LK17	LDUU00000000.1
Chromobacterium violaceum LK30	LK30	LDUX00000000.1
Chromobacterium violaceum LK6	LK6	LDUM00000000.1
Chromobacterium pseudoviolaceum LMG 3953	LMG3953	MQZX00000000.1
Chromobacterium subtsugae MWU12-2387	MWU122387	MQZZ00000000.1
Chromobacterium sp. MWU13-2610	MWU132610	PPTF00000000.1
Chromobacterium sp. MWU14-2602	MWU142602	PQWB00000000.1
Chromobacterium vaccinii MWU205	MWU205	JZJL00000000.1
Chromobacterium subtsugae MWU2387	MWU2387	LCWR00000000.1
Chromobacterium subtsugae MWU2576	MWU2576	LCWQ00000000.1
Chromobacterium subtsugae MWU2920	MWU2920	LCWP00000000.1
Chromobacterium vaccinii MWU328	MWU328	JZJJ00000000.1
Chromobacterium subtsugae MWU3535	MWU3525	LCWO00000000.1
Chromobacterium piscinae ND17	ND17	JTGE00000000.1
Chromobacterium subtsugae PRAA4-1	PRAA41	JYKA00000000.1
Chromobacterium haemolyticum T124	T124	JRFR00000000.1
